# Health System Capacity and Access Barriers to Diagnosis and Treatment of CVD and Diabetes in Nepal

**DOI:** 10.5334/gh.927

**Published:** 2021-05-18

**Authors:** Abhishek Sharma, Warren A. Kaplan, Gautam Satheesh, Indra Prasad Poudyal, Pawan Gyawali, Dinesh Neupane, Parash Mani Bhandari, Milan Malla, Surendra Sapkota, Shiva Raj Mishra

**Affiliations:** 1 Department of Global Health, Boston University School of Public Health, Boston, Massachusetts, US; 2 World Heart Federation, Salim Yusuf Emerging Leaders Progamme, Geneva, CH; 3 PRECISIONheor, Precision Value and Health, Boston, Massachusetts, US; 4 The George Institute for Global Health, Hyderabad, IN; 5 Tribhuvan University, Institute of Medicine, Kathmandu, NP; 6 Nepal Development Society, Bharatpur, Chitwan, NP; 7 Department of Epidemiology, Johns Hopkins Bloomberg School of Public Health, Baltimore, Maryland, US; 8 Department of Epidemiology, Biostatistics and Occupational Health, McGill University, Montreal, Quebec, CA; 9 Lady Davis Institute for Medical Research, Jewish General Hospital, Montreal, Quebec, CA; 10 Patan Academy of Health Sciences, Lalitpur, NP; 11 Department of Health Services, Ministry of Health and Population, Kathmandu, NP; 12 Faculty of Medicine, University of Queensland, Brisbane, AU

**Keywords:** cardiovascular disease, essential medicines, diagnostics, healthcare delivery, Sustainable Development Goals, Nepal

## Abstract

**Background:**

Universal access to essential medicines and routine diagnostics is required to combat the growing burden of cardiovascular disease (CVD) and diabetes. Evaluating health systems and various access dimensions availability, affordability, accessibility, acceptability, and quality is crucial yet rarely performed, especially in low- and middle-income countries.

**Objective:**

To evaluate health system capacity and barriers in accessing diagnostics and essential medicines for CVD and diabetes in Nepal.

**Methods:**

We conducted a WHO/HAI nationally-representative survey in 45 health-facilities (public-sector: 11; private-sector: 34) in Nepal to collect availability and price data for 21 essential medicines for treating CVD and diabetes, during MayJuly 2017. Data for 13 routine diagnostics was obtained in 12 health facilities. Medicines were considered unaffordable if the lowest paid worker spends >1 days wage to purchase a monthly supply. To evaluate accessibility, we conducted facility exit interviews among 636 CVD patients. Accessibility (e.g., private-public health facility mix, travel to hospital/pharmacy) and acceptability (i.e. Nepals adoption of WHO Essential Medicine List, and patient medication adherence) were summarized using descriptive statistics, and we conducted a systematic review of relevant literature. We did not evaluate medicine quality.

**Results:**

We found that mean availability of generic medicines is low (<50%) in both public and private sectors, and less than one-third medicines met WHOs availability target (80%). Mean (SD) availability of diagnostics was 73.1% (26.8%). Essential medicines appear locally unaffordable. On average, the lowest-paid worker would spend 1.03 (public-sector) and 1.26 (private-sector) days wages to purchase a monthly supply. For a person undergoing CVD secondary preventive-interventions in the private sector, the associated expenditure would be 7.511.2% of monthly household income. Exit-interviews suggest that a long/expensive commute to health-facilities and poor medicine affordability constrain access.

**Conclusions:**

This study highlights critical gaps in Nepals health system capacity to offer basic health services to CVD and diabetes patients, owing to low availability, poor affordability and accessibility of essential medicines and diagnostics. Research and policy initiatives are needed to ensure uninterrupted supply of affordable essential medicines and diagnostics.

## Introduction

Globally, non-communicable diseases (NCD) account for 71% of all deaths (i.e. estimated 41 of 57 million deaths in 2016) [1]. Cardiovascular diseases (CVDs) alone contribute to estimated 17.9 million deaths, accounting for 44% of NCD deaths and 31% of all-cause mortality [2]. Over three-quarters of the CVD deaths occur in low- and middle-income countries (LMICs), disproportionately affecting the poor households [3,4]. To tackle this burden, the United Nations Member States have pledged to reduce premature NCD deaths by a third by the year 2030 (Sustainable Development Goal; SDG 3.4) [5]. To achieve these goals, various international agencies including the World Health Organizations (WHO) 20132020 Global Action Plan and the World Heart Federations 25 25 vision are calling for ensuring availability of affordable essential medicines and health services in at least 80% of the health facilities in all sectors [6,7,8]. Essential medicines are those medicines that meet priority health needs of the majority of the population [9].

With its increasing NCD burden relative to infectious diseases, Nepals healthcare system now faces a dual challenge [10,11]. Most NCDs (e.g., CVD, diabetes) and their risk factors require lifelong treatment, often with multiple pharmaceutical agents, and this can amount to high cumulative healthcare costs. Nepal imports the majority of such pharmaceutical agents from neighboring India [12]. Moreover, CVDs and diabetes also require periodic use of diagnostic tests to inform treatment and ensure continuum of care [13]. With over a quarter of Nepals population living below the poverty line, people with chronic conditions face high risk of impoverishment due to catastrophic health expenditures [10,14]. Unfortunately, health insurance coverage in Nepal is still at nascent stage, which drives a majority of the patients to resort to out-of-pocket payments (OOP) [15,16].

It would, therefore, be important to evaluate access to essential medicines and diagnostics for treating CVDs and diabetes in Nepal [16,17,18]. The WHO and Health Action International (WHO/HAI) have developed a standardized methodology to measure the availability, prices and affordability of essential medicines [19,20]. Many WHO/HAI surveys conducted over the past decade have shown a major disparity between high and low income countries with respect to medicines access [21,22].

There is, nonetheless, no universally accepted definition of access to medicines, and various authors have suggested five access dimensions, namely: availability, affordability (both assayed using the WHO/HAI method), accessibility, acceptability (adoption), and medicine quality [16,23]. In brief, availability refers to the link between quantity required and quantity delivered. Affordability is the end-users ability to pay. Accessibility is an index of how easily a person can get the medicine and is a function of distance traveled as well as operational logistics of the medicine dispensary, among other factors. Acceptability/adoption is a complex function of the kind of, and manner in which, medicines are prescribed by physicians, dispensed by pharmacists, and actually used by patients (e.g. treatment adherence). Quality is based on empirical standards created by the relevant medicines regulatory authority. The literature notes the need for relatively inexpensive and rapid methods to collect such important information repeatedly that allows for the integration of multiple access dimensions including indirect costs as well as indication of the potential impact of survey price on household income [16,24].

A recent critique of Nepals current disease-centred vertical health programs included a plea to reform and regulate the procurement of medicines, medical equipment, and supplies to set minimum quality criteria and reduce costs, implicating indirectly the dimensions of availability and affordability [25]. Yet, the few surveys that have actually explored the dimensions of access of essential medicines in Nepal were either not nationally-representative or were not CVD-specific. None of these studies evaluated access to CVD diagnostics, which are essential for tackling the NCD disease burden (**Box 1**). We therefore investigated various dimensions of access (except medicine quality) to essential CVD and diabetes medicines and diagnostics in a nationally-representative sample in Nepal, and explored barriers that limit access to those basic health services.

**Box 1: KEY MESSAGES.**

**What is already known about this subject?**

- The United Nations Member States, including Nepal, have pledged to improve access to essential health services aiming to reducing premature non-communicable disease deaths by 30% by 2030. In Nepal, limited access to essential medicines (EMs) and diagnostics for CVD and diabetes is a major public health concern, where these conditions disproportionately affect the poor.
- In low- and middle-income countries, surveys have examined the availability of medicines for different diseases in hospitals or clinics. However, little is known about the various dimensions of access to CVD diagnostics and medicines.
- The definition of access to medicines includes multiple dimensions, and most evaluations equate only price and physical availability as the primary proxies for access. Our literature review found that the majority of access studies in Nepal focused on medicine availability and affordability, and only a few commented on the barriers to access, local adoption of EMs, or access to essential diagnostics. None of these access studies measured the impact of CVD management costs on household income or systematically measured multiple dimensions of access in a nationally-representative sample (Appendix E Table 1).

**What does this study add?**

- In our nationally-representative survey looking at different dimensions of access, the mean availability of CVD and diabetes EMs (<50%) and diagnostics (<75%) in Nepal fell short of the WHOs 80% availability target. In the public sector, only 6 out of 13 surveyed diagnostic tests were available in at least 80% of the facilities. The private-sector EMs and diagnostics appear locally unaffordable. Health system capacity for delivering care is further limited by difficulty faced by patients in actually getting to the point-of-care.
- The financial impact of managing CVD could be significant as private sector-based secondary prevention management of CVD for an individual can yield expenditures between 7.511.2% of monthly household income.
- Nepals essential medicines list (EML) contains most of the WHO-recommended medicines. However, some effective and cheaper options such as the recently approved anti-hypertensive fixed-dose combinations are missing from Nepals EML.

**What are the implications for clinical practice and health policy?**

- The existing Nepalese healthcare system is not well prepared to offer basic health services to CVD and diabetes patients. Nepal needs to develop initiatives to ensure uninterrupted supplies of affordable diagnostics and EMs. Nepal cannot locally produce these medical commodities, so this would require scaling up the ability of local bodies to manage medicine procurement and general logistics, regularly updating the EML, and having adequate human resources in local healthcare centers.

## Methods

We employed a mixed-methods approach. We used a modified version of the WHO/HAI methodology, during MayAugust 2017, to collect availability and price data in public and private healthcare sectors. A typical WHO/HAI survey is limited to a pre-defined list of core global medicines along with medicines selected by the investigator, but we also surveyed access to routinely-used diagnostics.

In addition, we conducted exit interviews with patients at healthcare facilities, using a semi-structured questionnaire, to understand additional access dimensions for example, an individuals ability to access a health facility and obtain prescribed medicines [16]. To measure acceptability/adoption, we collected information on patient adherence measures in exit interviews, and conducted a desk review to compare medicines included in the latest Nepals Essential Medicine List (EML) with the WHO recommendations.

### Sampling

#### Survey Facilities

**Figure 1:**
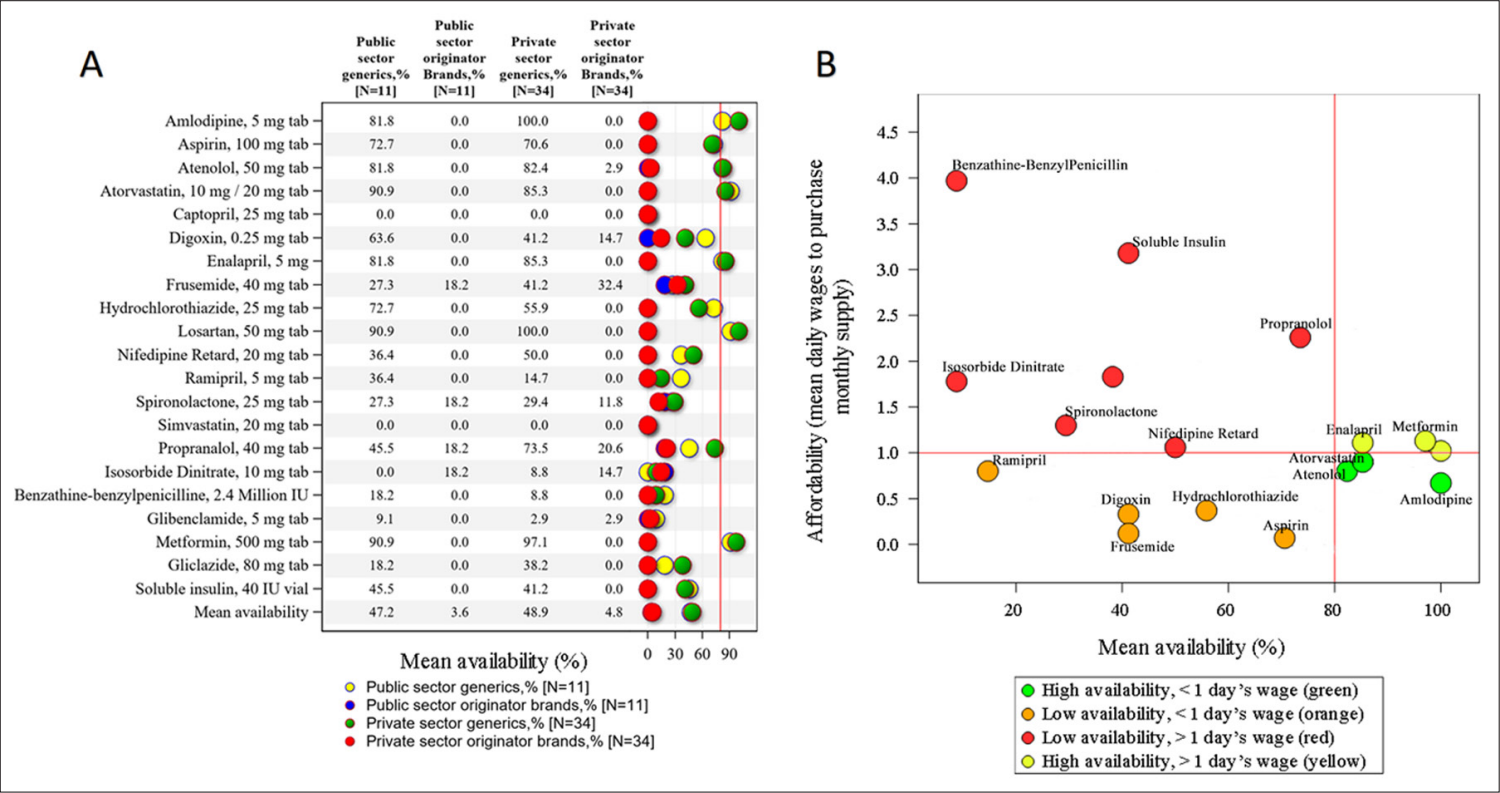
**(A)** Mean availability of CVD and diabetes essential medicines in Nepals public and private sectors, and **(B)** Availability and affordability of selected medicines in the private-sector facilities.

Nepal has a population of ~29 million, the majority being concentrated in the capital, Kathmandu. We selected Kathmandu as the central survey area. Seven additional districts namely, Bhaktapur, Rupandehi, Ilam, Jhapa, Khotang, Syangja and Kailali were selected, representing five out of Nepals seven administrative divisions, i.e., all provinces except province no. 2 and 6. (Appendix E Figure 1).

We obtained a list of all public-sector hospitals in each of the selected districts. In each of the survey district, we randomly selected at least one public-sector hospital as survey anchor, for a total of 11 public-sector hospitals. Within two km radius of the 11 survey anchor facilities, we surveyed a total of 34 private-sector retail pharmacies. To collect data on routine CVD diagnostic tests, we surveyed a total of 12 secondary/tertiary care hospital facilities (nine in the public sector and three in the private sector) among the survey districts, during MayJuly 2017.

#### Survey Medicines

Upon reviewing the WHO Model EML, Nepals 2011 and 2016 National EMLs, and guidance documents on essential CVD care [13,26], we identified 21 CVD essential medicines and 13 diagnostic tests for the survey (Tables 1,2).

**Table 1:** Median price ratios (MPR) and affordability of generic essential medicines in Nepal.

	Generic name, dosage form, strength	2015 MSH IRPs (USD)	Public Sector		Private Sector		% price increase in private sector compared to public sector

			MPR (Ratio of Median consumer price to MSH IRP)	Number of days wages for monthly supply	MPR (Ratio of Median consumer price to MSH IRP)	Number of days wages for monthly supply	

1	Amlodipine, *5 mg tab*	0.0061	6.38*	0.57	7.47*	0.67	17.3%
2	Aspirin, *100 mg tab*	0.0062	0.72	0.07	0.77	0.07	7.5%
3	Atenolol, *50 mg tab*	0.0059	5.49*	0.71	6.11*	0.80	11.4%
4	Atorvastatin, *10 mg/20 mg tab*	0.0233	3.14	1.08	2.63	0.90	16.3%
5	Benzathine-benzylpenicilline, *2.4 million IU*	0.2254	N/A	N/A	0.79	3.97	4.7%
6	Captopril, *25 mg tab*	0.0076	N/A	N/A	N/A	N/A	N/A
7	Digoxin, *0.25 mg tab*	0.0169	1.42	0.35	1.33	0.33	6.0%
8	Enalapril, *5 mg*	0.0062	4.64*	0.85	6.08*	1.11	31.0%
9	Frusemide, *40 mg tab*	0.0062	1.24	0.11	1.37	0.13	10.5%
10	Glibenclamide, *5 mg tab*	0.0053	14.79*	2.31	N/A	N/A	N/A
11	Gliclazide, *80 mg tab*	0.0222	1.98	1.30	2.79	1.83	41.5%
12	Hydrochlorothiazide, *25 mg tab*	0.0049	5.43*	0.39	5.09*	0.37	6.3%
13	Isosorbide Dinitrate, *10 mg tab*	0.0215	N/A	N/A	0.94	1.78	N/A
14	Losartan, *50 mg tab*	0.0181	3.01	0.80	3.83	1.02	27.2%
15	Metformin, *500 mg tab*	0.0162	0.89	0.85	1.18	1.13	33.3%
16	Nifedipine Retard, *20 mg tab*	0.3840	0.11	0.90	0.13	1.06	17.8%
17	Propranalol, *40 mg tab*	0.0108	2.42	1.54	3.55	2.26	47.1%
18	Ramipril, *5 mg tab*	N/A	N/A	0.69	N/A	0.80	16.5%
19	Simvastatin, *20 mg tab*	0.0163	N/A	N/A	N/A	N/A	N/A
20	Soluble insulin, *40 IU vial*	4.3800	0.47	3.02	0.49	3.18	5.2%
21	Spironolactone, *25 mg tab*	0.0442	0.72	1.40	0.66	1.30	7.3%
**Median MPR [range]**			**2.19 [0.1114.79]**		**1.37 [0.137.47]**		**Mean: 13.8%**
**Mean (SD) [Median (range)] number of daily wages for monthly supply**			**1.03 (0.77) [0.85 (0.073.02)]**		**1.26 (1.03)[1.04 (0.073.97)]**		

Median Price Ratio (MPR) is calculated by dividing the median consumer price of a given medicine with the respective MSH international reference price (IRP). An MPR of 1.00 would mean that the medicine consumer price is equal to its IRP. The WHO recommends that median consumer price should not be 4 times greater than the MSH IRP. MRPs greater than 4.00 are marked with asterisks (*). All unaffordable medicines (i.e. those medicines for which a months supply costs > 1 days lowest paid wage) are marked with symbol (). N/A refers to the medicines where MSH IRP or at least four consumer price data points were not available.

**Table 2:** Availability, prices and affordability of routine CVD and diabetes diagnostic tests in secondary and tertiary healthcare facilities in Nepal.

Name of the diagnostics	Availability (%)			Median Price of single test (USD)		No. of days wages for a single test	

	Public Sector (N = 9)	Private Sector(N = 3)	Overall	Public Sector	Private Sector	Public Sector	Private Sector

Creatinine	100.0%	100.0%	100.0%	0.96	1.92	0.47	0.94
ECG	77.8%	66.7%	75.0%	1.92	2.88	0.94	1.41
Full Blood Count	88.9%	100.0%	91.7%	1.39	1.92	0.68	0.94
Glycemia	100.0%	100.0%	100.0%	0.58	0.96	0.28	0.47
HbA1c	55.6%	33.3%	50.0%	3.84	0.96	1.88	0.47
HDL cholesterol	55.6%	66.7%	58.3%	1.92	1.68	0.94	0.82
Kalemia	55.6%	66.7%	58.3%	1.34	2.64	0.66	1.29
LDL Cholesterol	11.1%	0.0%	8.3%				
Proteinurea	88.9%	100.0%	91.7%	0.34	0.96	0.16	0.47
Total Cholesterol	66.7%	66.7%	66.7%	1.44	1.68	0.71	0.82
Triglyceride	55.6%	66.7%	58.3%	0.96	1.68	0.47	0.82
Urea	100.0%	100.0%	100.0%	0.96	1.92	0.47	0.94
Uric Acid	88.9%	100.0%	91.7%	0.94	1.92	0.46	0.94
**Mean**	**72.6%**	**74.4%**	**73.1%**			**0.68 days**	**0.86 days**

### Data Collection & Analysis

Upon visiting the survey pharmacy facilities, the data collectors first explained the purpose of the study to the pharmacy personnel. Data collectors assessed product availability (in stock) on the day of survey and then collected information, including consumer price data for the originator brand (OB) and the lowest priced generic (LPG) versions of each survey medicine.

We report availability as the percentage of surveyed facilities where a given medicine/diagnostic test was found on the day of survey. We evaluated consumer prices using the Management Sciences for Health international reference prices (MSH IRPs) as an external benchmark [27]. The MSH IRPs are the procurement prices obtained by government agencies, pharmaceutical suppliers and international development organizations, and are widely accepted as an appropriate reference standard. For medicine price analysis, we compared consumer prices in Nepal with the 2015 MSH IRPs to calculate medicine-specific median price ratios (MPRs). According to the WHO, no patient should pay more than four times the IRPs. We also determined price variation among sectors i.e. percentage difference in price of CVD medicines in the private and public sector facilities. We also conducted medicine affordability analysis [19], where a chronic medicine is considered unaffordable if the lowest-paid worker has to spend over one days wage (i.e. 212.5 Nepalese rupees (NPR)/2.039 USD) [28] to purchase a one month medicine supply. For each CVD diagnostic test, we report median unit consumer price and how that compares to the lowest paid workers daily wage.

To evaluate medicine accessibility, during hypertension screening for the 2017 May Measurement Month [29], we conducted exit interviews, using a semi-structured questionnaire, among 949 patients (aged 18 years or above) who were visiting for regular health check-up and were found to be hypertensive. Of these 949 patients, we analyzed access-related data obtained from patients who were clinically diagnosed with and/or were prescribed medicines for CVD or diabetes. Alongside reporting various access barriers (such as travel time to health/medicine facility, public-private health sector mix) among surveyed patients, we estimated how much the medicines and diagnostic tests required for managing various CVD risk profiles in Nepals public and private sector would cost as proportion of the patients monthly household income. Medication adherence (a measure of acceptability) was assessed using the 4-item Morisky Green Levine (MGL) medication adherence scale, a validated and widely-used tool that asks short behavioral questions in such a way that helps avoid yes-saying bias commonly observed in chronic care patients [30,31].

## Results

### Availability of Surveyed Essential Medicines

Figure 1A summarizes the mean availability of the surveyed CVD essential medicines stratified by OB and generic equivalents in Nepals public and private sectors.

The mean public and private sector availability of generic versions of CVD medicines was 47.2% and 48.9%, respectively. In both sectors, only 28.6% (n = 6) of the surveyed medicines met the WHOs 80% availability target; these included amlodipine, atenolol, atorvastatin, enalapril, losartan and metformin. Captopril and simvastatin were not available in any surveyed facility.

The mean availability of OB versions in the public and private sectors was 3.6% and 4.8%, respectively. We found OB versions of only four medicines (4/21 = 19.1%) in the public sector and seven medicines (7/21 = 33.3%) in the private sector. In both the sectors, mean availability of the OB version of isosorbide dinitrate was higher than its generic counterpart but was no higher than 20%.

### Price and Affordability of Surveyed Essential Medicines

Table 1 summarizes the availability and affordability of generic CVD EMs, stratified by sector. Median MPR of CVD EMs was 2.19 [range: 0.11 (nifedipine) 14.79 (glibenclamide)] in the public-sector and 1.37 [0.13 (nifedipine) 7.47 (amlodipine)] in the private-sector facilities. Compared to the public sector, on average, private-sector prices were 13.8% higher and those for enalapril, glicazide, losartan, metformin and propranalol were over 25% higher.

In Nepals private sector, a lowest paid worker would spend between the range of 0.07 (aspirin) and over three days wages (insulin and benzathine-benzyl penicillin) to purchase a monthly supply of a given generic CVD/diabetes EM. On average, a monthly supply of any individual generic EM would cost 1.03 and 1.26 days wages in the public and private sectors, respectively. In the public and private sectors, a monthly supply of nearly 29% and 50% of the surveyed medicines, respectively, would cost more than a days wage, for a monthly supply. See Appendix E Table 2 for median price of OB medicines.

Figure 1B plots affordability against availability for private-sector generic medicines. The most accessible are those lying at or near the lower right-hand quadrant, that is, enalapril, metformin, atorvastatin, atenolol and amlodipine.

### Availability and Affordability of Surveyed Diagnostic tests

Table 2 summarizes the availability, prices and affordability of routine diagnostic tests in Nepals public and private sectors. In both sectors combined, mean (SD) availability was 73.1% (26.8%); ranging from 8.3% (LDL cholesterol) to 100% (creatinine and urea). Mean availability of diagnostics was 74.4 and 72.6 in the private and public sectors, respectively. Tests for LDL cholesterol were not available in any of the three surveyed private-sector hospitals.

In the public sector, the median price of diagnostic tests ranged from USD 0.34 (proteinuria) to USD 3.84 (HbA1c). The median public-sector prices of all diagnostic tests except HbA1c and HDL cholesterol were lower compared to the private sector. In the private sector, the median price of diagnostic tests ranged from USD 0.96 to USD 1.92. The lowest-paid worker would spend mean 0.68 [range: 0.16 (proteinurea) 1.88 (Hb1Ac)] and 0.86 [range: 0.47 (proteinurea) to 1.41 (ECG)] days wage to pay for a single diagnostic test in the public and private sectors, respectively.

Table 3a and 3b show the estimated monthly costs of managing different cardiovascular risk profiles in Nepals private and public sectors, respectively, as proportion of three monthly-household-income levels i.e. USD 100 ( NPR 10,000), USD 100 200, and USD 200 300. See appendix E Table 3 for distribution of surveyed patients monthly household income. At the lowest CVD risk that requires only annual risk monitoring, the costs for such management as a proportion of monthly household income of USD 100 would be 0.57% and 0.43% in the private and public sectors, respectively. As the risk profiles increase for primary prevention, the estimated proportions also increase. The ranges are based on the least and most expensive of the various medicines listed. Thus, a high CVD-risk person who belongs to the lowest household income level (100 USD/month) and undergoes private-sector interventions for primary prevention, would spend 5.06.6% of their monthly household income on these interventions. If this person is undergoing clinical CVD interventions as secondary prevention, the expenditure would be 7.511.2% of the monthly household income.

**Table 3:** Estimated costs of managing cardiovascular risk profile in Nepals private and public sectors.

3A. Private sector								

Prevention	Risk	Intervention	Cost of medicines (USD | no. of days wages)	Cost of tests(USD | no. of days wages)	Total cost (USD | no. of days wages)	Total cost as proportion of monthly household income		

						USD 100	USD 100 200	USD 200 300

**Primary**	<10%	Lifestyle changes + risk monitoring once in 12 months	N/A	0.571 | 0.28	0.571 | 0.28	0.57%	0.29%	0.19%
	1020%	Lifestyle changes + risk monitoring once in 6 months	N/A	1.162 | 0.57	1.162 | 0.57	1.16%	0.58%	0.39%
	2030%	Statin^a^ + one antihypertensive^b^ + risk monitoring once in 6 months	2.5904.098 | 1.272.01	1.162 | 0.57	3.7525.260 | 1.842.58	3.755.26%	1.882.63%	1.251.75%
	30%	Statin^a^ + one antihypertensive^b^ + aspirin + risk monitoring once in 3 months	2.7324.241 | 1.342.08	2.304 | 1.13	5.0366.545 | 2.473.21	5.046.55%	2.523.27%	1.682.18%
**Secondary**		-blocker^c^ + ACE Inhibitor^d^ + statin ^a^ + aspirin + risk monitoring once in 3 months	5.2408.849 | 2.574.34	2.304 |1.13	7.54411.153 | 3.705.47	7.5411.15%	3.775.58%	2.513.72%
**3B. Public sector**								

**Primary**	<10%	Lifestyle changes + risk monitoring once in 12 months	N/A	0.428 | 0.21	0.428 | 0.21	0.43%	0.21%	0.14%
	1020%	Lifestyle changes + risk monitoring once in 6 months	N/A	0.877 | 0.43	0.877 | 0.43	0.88%	0.44%	0.29%
	2030%	Statin^a^ + one antihypertensive^b^ + risk monitoring once in 6 months	2.9974.037 | 1.471.98	0.877 | 0.43	3.8744.914 | 1.902.41	3.874.91%	1.942.46%	1.291.64%
	30%	Statin^a^ + one antihypertensive ^b^ + aspirin + risk monitoring once in 3 months	3.1404.180 | 1.542.05	1.733 | 0.85	4.8735.913 | 2.392.90	4.875.91%	2.442.96%	1.621.97%
**Secondary**		-blocker^c^ + ACE Inhibitor^d^ + statin^a^ + aspirin + risk monitoring once in 3 months	5.1997.218 | 2.553.54	1.733 | 0.85	6.9328.950 | 3.404.39	6.938.95%	3.474.48%	2.312.98%

Minimal WHO Recommended tests (fasting blood sugar, cholesterol, potassium levels, proteinuria and ECG) would cost the lowest paid worker 6.10 and 4.16 days wages in the private and public sectors respectively. Risk monitoring (lipid profile, fasting blood sugar and proteinuria) would cost 3.40 and 2.56 days wages in private and public sectors respectively. From these values, we calculated costs for monitoring risk once in 12 months, 6 months and 3 months. ^a^ Includes atorvastatin and simvastatin; ^b^ Includes amlodipine, nifedipine, hydrochlorothiazide, ramipril, captopril and enalapril; ^c^ Includes atenolol and propranolol; ^d^ Includes ramipril, captopril and enalapril. Lowest daily wage for workers in Nepal at the time of survey was USD 2.039 (NPR 212.5). Mean household income among the exit interview participants was USD 239.87 (NPR 25,000). Diabetic patients would require a hypoglycaemic medicine (metformin/insulin) which additionally costs 1.133.18 days wages in private sector and 0.853.02 days wages in public sector.

We note that if this same person were unfortunate enough to also suffer from diabetes and require either metformin or insulin, the private-sector cost for these medicines would lead to an additional 2.3% (metformin) to 6.5% (insulin) of USD 100 monthly household income, 1.2%3.2% with USD 200 monthly income, and 0.8%2.3% with USD 300 monthly income. The highest percentage of monthly household income spent by one person in a household undergoing insulin treatment as well as secondary prevention for CVD would approach 18% (i.e., 11.2% + 6.5%).

### Accessibility to healthcare/pharmacy facility

Of the 949 patients, 67.0% (n = 636) had been clinically diagnosed with and/or were prescribed medicines for CVD or diabetes but only 56.0% (n = 531) of them were prescribed anti-hypertensive medications.

The 636 patients with confirmed evidence of CVD and/or diabetes were interviewed for access barriers. Among those, the median (Q1-Q3) age was 55.0 (44.064.0) years, 51% were women, and 56% of patients had a household income of NPR 20,000 (USD 167.7) or higher. See Appendix E Table 3 for demographic details. The majority (n = 328; 51.1%) sought general healthcare advice in the private sector. Forty-four patients (6.9%) reported to have missed a scheduled healthcare consultation in the last month. A majority of patients reported purchasing medicines in the private sector (retail pharmacies: 16.7%; hospital/clinics: 56.8%), and 26.6% obtained medicines from public-sector hospitals or primary healthcare centers. Twenty-nine patients (4.6%) reported to not have any prescribed medicines at home, and most of these interviewees (82.8%) reported an inability to afford medicines as the main reason limiting access. A majority of patients walked to their respective health facility (53.9%), followed by use of a bicycle or motor vehicle (e.g., private vehicle, public bus). Patients spent a median of 15, 20 and 60 minutes, respectively, via walk, bicycle and motor vehicle. Patients who traveled to health facility by motor vehicle spent about NPR 100 (USD 0.96) per visit. Furthermore, about 25.9% patients used alternative treatment/therapy to manage their cardio-metabolic diseases along with physician advice and prescribed allopathic medications. Of these, most patients used Ayurvedic and Homeopathic medicines. See Table 4.

**Table 4:** Medicine accessibility among CVD and diabetes patients.

Access measures	Number of patients (%)		

Patients who were diagnosed with and/or prescribed medications for CVD or diabetes (cardio-metabolic).	636 (100.0%)		
Patients who already had been prescribed with anti-hypertensive medication.	531 (83.5%)		
**Healthcare checkup and consultation by sector, n (%)**			
Distribution of healthcare facility-mix where patients sought regular consultations and advice.			
Public sector	106 (16.7%)		
Private sector	205 (32.2%)		
Both	328 (51.1%)		
Patients who missed a scheduled healthcare visit in last one month.	44 (6.9%)		
**Medication use and access, n (%)**			
Patients who were diagnosed with and/or prescribed medications for CVD or diabetes (cardio-metabolic).	636 (100.0%)		
Outlets where patients usually obtained their medications.			
Public-sector hospitals	115 (18.1%)		
Public-sector primary health care centers	54 (8.5%)		
Private-sector hospitals/clinics	361 (56.8%)		
Private retail pharmacies	106 (16.7%)		
Patients who did not have prescribed cardio-metabolic medications at home, n (%).	29 (4.6%)		
Reasons reported for limited access (i.e. no medicines at home).			
Medicines unavailable at pharmacy facility.	1 (3.5%)		
Medicine available but not affordable.	24 (82.8%)		
Lack of time to purchase medicines.	4 (13.8%)		
Travel to healthcare/pharmacy facility to obtain medications.	n (% patients)	Time in minutes, Median (IQR)	Cost in NPR, Median (IQR)
Mode of Transportation.			

Walk	342 (53.9%)	15 (10, 30)	
Cycle	16 (2.5%)	20 (10, 30)	
Motor vehicle (Bus/car/taxi)	276 (9.5%)	60 (30, 60)	100 (40, 150)
Patients who also use alternative therapy, apart from physician/allopathic treatment, n (%).	165 (25.9%)		
Ayurveda	23 (3.6%)		
Homeopathy	15 (2.4%)		
Salam healers/dhami/Jhakri	3 (0.5%)		
Home remedies	109 (17.1%)		
Others	15 (2.4%)		
Medication adherence			
Morisky predictive score*, Mean (SD)	2.9 (0.46)		
Patients who ever forget to take medicine on time, n (%)	71 (11.2%)		
Patients who reported to be careless about taking medicines, n (%)	604 (95.4%)		
Patients who sometime stop taking medicines when they feel better, n (%)	30 (4.7%)		
Patients who sometime stop taking medicines when they feel worse, n (%)	17 (2.7%)		

* Adherence level on a scale of 04, where score 0 refers to lowest and score 4 refers to highest level of adherence.

### Acceptability (adoption)

Nepals national EML was last updated in 2016, five years after the prior version. The 2016 Nepal list includes a total of 36 CVD and diabetes medicines (irrespective of dosage form). These include 25 out of 30 medicines listed in the 2017 WHO Global EML (83.3%), and of 35 medicines listed in the 2019 WHO Global EML (71.4%). Nepals 2016 EML has 10 medicines [i.e. oral formulations of disopyramide, fenofibrate, glipizide, nifedipine, prazosin, procainamide and ramipril, and injections of dobutamine, isoprenaline, labetalol and procainamide] that are not listed in either the 2017 or 2019 WHO Global EML. Furthermore, the WHO approved four BP-lowering fixed-dose combinations (FDCs) for inclusion in the 2019 Global EML, and these FDCs are not listed in the Nepalese 2016 EML [32,33]. See details in Appendix E Table 4. In another measure of acceptability, we found that medication adherence measured using the MGL Adherence Scale was poor among the surveyed population (Table 4).

## Discussion

In Nepal, CVDs are the leading cause of lost disability-adjusted life years and mortality [34,35], and the nation-wide prevalence of type 2 diabetes is 8.4% (95% CI: 6.210.5%) [36], approaching 12% in semi-urban areas [37]. To the best of our knowledge, this is the first study evaluating the preparedness to deliver NCD care in Nepal, in terms of availability, prices, affordability and accessibility of both medicines and diagnostics that are essential to treat CVDs and diabetes. The risk factors for CVD and diabetes begin sub-clinically and patients may not present to healthcare professionals until there are serious symptoms making early diagnosis and disease management difficult [38]. There are multiple reasons for such late patient presentation but limited access to both medicines and diagnostics is surely one of them. We note that both CVD and diabetes are, unfortunately, underlying conditions contributing to COVID-19 mortality [39].

Mean availability of generic versions of surveyed medicines is low (<50%) in both public and private sectors, and less than one third of the surveyed medicines met the WHOs 80% availability target. Mean availability of OB versions was nearly ten times lower than their generic counterparts, which is not surprising as price competition from generics has essentially eliminated OB in most LMIC markets [40]. Our findings agree with the few, earlier assessments that reported overall limited availability of medicines, and that the availability is relatively higher in the private sector (Appendix E Table 1). A recent survey of NCD medicines in Nepal reported higher availability estimates (70% in private sector vs. 68% in public sector) compared to our findings, possibly because the 2015 survey was not CVD or diabetes-focused and collected data from pharmacy facilities only in highly-populated areas [41].

Notwithstanding the WHO recommendation that no medicine should cost more than four times its IRP, five surveyed essential medicines (amlodipine, atenolol, enalapril, glibenclamide and hydrochlorothiazide) exceeded this target, although the IRP for these medicines is very low (See Figure 1b). The monthly supply of aspirin, a statin, a beta-blocker, and ACE inhibitor cost the lowest paid worker about 2.6 to 4.3 days wages in Nepals private sector, depending on the drugs prescribed. Although monotherapy with first-line agents for hypertension (i.e. hydrochlorothiazide or amlodipine) was affordable, most hypertensive patients eventually require combination therapy with other agents, which may well be difficult to afford. However, monotherapy with oral hypoglycemic agents (e.g., glibenclamide, gliclazide, metformin) or insulin are expensive in both sectors, costing between 0.85 and 3.18 days wages. This is consistent with prior findings [41,42]. The majority (82.8%) of exit survey participants reported their general inability to afford medicines as the main reason limiting access. Several of the surveyed medicines (over 35% in public sector and 50% in private sector) cost more than 1-days wage (Table 1). One measure towards reducing cost of medicines would be to update the Nepalese EML and clinical guidelines to adopt BP-lowering FDCs that were recently included in the 2019 WHO EML based on their clinical efficacy and lower costs [32,33].

Medicines to treat CVD and diabetes are only part of the story. Diagnostic tests are essential to provide effective care to patients. Not only are those required for early identification of disease, but they also can ensure appropriate therapy initiation and continued disease and risk monitoring. In Nepals public and private sectors, mean availability of surveyed diagnostic tests ranged from 8.3% (LDL cholesterol) to 100% (creatinine and urea). An overall lack of health facilities diagnostics capacity limits a health systems preparedness to provide general health services related to NCD care [4345]. Although diagnostics appear affordable in our study, over-expenditure is likely in patients with higher health risk who require frequent risk-monitoring. Regarding other dimensions of access, we found that the majority of patients walked to their respective health facility, the rest coming by bicycle or motorized transportation. Long motorized travel times averaging one hour are indicative of far-off health facilities, and erratic schedules and wait times in public-transport.

Expensive medicines and diagnostics may impose a significant household financial burden with high out-of-pocket (OOP) healthcare payments leading to financial distress [46]. We estimated the fraction of monthly income relegated to medicines and diagnostic tests under certain assumptions of CVD risk. This fraction can range upwards of 10% of monthly household income for one person with CVD in a Nepalese household (See Tables 3a and 3b). There exists even a higher risk for catastrophic health spending when CVD patients also suffer from diabetes, a not uncommon occurrence [47]. A majority of the Nepal population resorts to out-of-pocket (OOP) payments, which constitute nearly 75% of the overall private expenditure much larger than the WHO estimate of 45% OOP expenditure in LMICs [14].

In Nepal, the EML only serves as a guide for health practitioners; there is a lack of financial commitment from the government to provide the medicines and vaccines on the list, which means that they may not necessarily be free of charge or even available in public facilities [48]. Nepals EML implementation suffers from a weak evidence-based foundation sufficient to develop EMLs that are verifiable and accurate [48]. We urge that evidence-based criteria should be implemented for proper EML resource allocation, including medicine and diagnostic procurement and distribution, as it is important to outline a mechanism for its development. The health technology assessment model proposed by Singh, et al. (2017) offers a useful starting point [48].

Over the past decade, Nepal has introduced some programs to prioritize provision of essential healthcare services with special emphasis on CVDs. Their implementation requires knowledge of NCD burden, which is impossible without a national registry system to monitor NCDs. Nepal recently underwent a transition to federalism, aiming to decentralize political and economic power [25,49]. Within the first two years of the transition, provincial and local governments accounted for about 34% of the national budget, significantly boosting their fiscal responsibility [50]. In April 2016, Nepal rolled out its National Health Insurance Programme (NHIP) that offers health services to those enrolled on voluntary basis enrollees through public-sector and select private-sector facilities. Despite NHIPs subsidized premium rates for the socio-economically disadvantaged, its adoption remains low and cited reasons include limited access to services [51,52]. Ensuring uninterrupted supplies of medical commodities and services is a public health challenge, as Nepal is not a major local producer of medicines [12]. This requires scaling up the ability of local bodies to manage drug procurement and general logistics and have adequate human resources in local healthcare centers [25,49]. This scale-up provides the opportunity to embed improvement in access dimensions of medicines and diagnostics directly in the core of health system policy and planning [25].

## Limitations

The WHO/HAI survey is cross-sectional and misses the patterns of access over time. We could not perform a household survey which would tally all household expenses. Such a survey is, at present, the best way to really understand the poverty-inducing impact of biomedical product prices on household income. Further, Nepalese essential medicine lists are not continually updated, which results in obsolete medicines such as captopril being listed as essential. Our study did not evaluate the quality dimension of access due to lack of required resources. Lastly, the patients in our exit surveys were interviewed at healthcare facilities and these patients may actually have better care-seeking behavior and/or improved access to healthcare than the average population.

## Conclusions

Access to quality and affordable medicines are pivotal to achieving universal health coverage in Nepal. Our study suggests that much needs to be done in several dimensions of access to CVD and diabetes care. Particularly at the local level, information on access to medicines is necessary to make data-driven decision regarding optimizing delivery of health services. For Nepal, increasing access to health services, improving quality of health services and quality of affordable medicines has not received much attention. The National Insurance Policy of 2013, and The National Health Sector Strategy (20152020) was built on a patchwork of global policies not entirely based on local needs and precludes a strong focus on establishing a national medicines price monitoring system. Thus, establishing a national drug price monitoring body may improve procurement. Further regular mapping of access to medicines, by leveraging the large national surveys, could potentially help to continuously monitor the situation until more efficient monitoring systems are developed. On the global level, such efforts can leverage into outreach programs like International Society of Hypertensions flagship blood-pressure screening campaign [53].

This study provides evidence of limited access to cardiovascular and diabetes care in both public and private sectors of Nepal due to low availability and poor affordability of essential medicines and diagnostic tests. Research and policy efforts are needed to improve access.

## Data Accessibility Statement

The data used and/or analyzed for the current study are available from the corresponding authors on reasonable request.

## Additional File

The additional file for this article can be found as follows:

10.5334/gh.927.s1 Appendix. Supplementary Online Material.

## References

[1] World Health Organization. Global Health Estimates 2016: Deaths by Cause, Age, Sex, by Country and by Region, 20002016 [Internet]. Geneva: World Health Organization; 2018. Retrieved from: https://www.who.int/healthinfo/global_burden_disease/GHE2016_Deaths_WBInc_2000_2016.xls.

[2] World Health Organization. Noncommunicable diseases country profiles 2018. Geneva: World Health Organization; 2018. Licence: CC BY-NC-SA 3.0 IGO.

[3] World Health Organization. Cardiovascular Diseases (CVDs) [Internet]. Retrieved from https://www.who.int/en/news-room/fact-sheets/detail/cardiovascular-diseases-(cvds) (accessed 12 January 2021).

[4] World Health Organization. Global Action Plan for the Prevention and Control of Noncommunicable Diseases 20132020. Geneva: World Health Organization; 2013.

[5] Department of Economic and Social Affairs. Sustainable Development Goals: Transforming Our World: The 2030 Agenda for Sustainable Development. New York: United Nations; A/RES70/1. Retrieved from https://sdgs.un.org/goals (accessed 12 December 2020).

[6] Yusuf S, Wood D, Ralston J, Reddy KS. The World Heart Federations vision for worldwide cardiovascular disease prevention. Lancet. 2015; 386(9991): 399 402. DOI: 10.1016/S0140-6736(15)60265-3 25892680

[7] World Health Organization. Global Action Plan for the Prevention and Control of Noncommunicable Diseases 20132020. Geneva: World Health Organization; 2013.

[8] World Health Organization. Methodology to measure access to medicines for Sustainable Development Goal Indicator SDG 3.b.3. Geneva: World Health Organization; 2018. Retrieved from: https://www.who.int/medicines/areas/policy/monitoring/methodology_access_medicines_SDG_3_b_3/en/ (accessed 17 April 2021).

[9] Sharma A, Rorden L, Ewen M, Laing R. Evaluating availability and price of essential medicines in Boston area (Massachusetts, USA) using WHO/HAI methodology. J of Pharm Policy and Pract. 2016; 9: 12. DOI: 10.1186/s40545-016-0059-5 27054040 PMC4822245

[10] Asian Development Bank. [Internet]. Manila: The Bank 2010. Poverty Data: Nepal. Retrieved from www.adb.org/countries/nepal/poverty (24 Feburary 2020).

[11] Mishra SR, Neupane D, Bhandari PM, Khanal V, Kallestrup P. Burgeoning burden of non-communicable diseases in Nepal: a scoping review. Global Health. 2015; 11: 32. DOI: 10.1186/s12992-015-0119-7 26178459 PMC4504073

[12] Sharma A, Mishra SR, Kaplan WA. Trade in medicines and the publics health: A time series analysis of import disruptions during the 2015 India-Nepal border blockade. Global Health. 2017; 13: 61. DOI: 10.1186/s12992-017-0282-0 28830500 PMC5568715

[13] World Health Organization. New Essential Medicines and Diagnostics Lists published today [Internet]. Geneva: The Organization; 2019. Retrieved from www.who.int/medicines/news/2019/updates-global-guidance-on-medicines-and-diagnostic-tests/en/ (accessed 25 June 2020).

[14] Saito E, Gilmour S, Rahman MM, Gautam GS, Shrestha PK, Shibuya K. Catastrophic household expenditure on health in Nepal: A cross-sectional survey. Bull. World Health Organization. 2014; 92(10): 760 7. DOI: 10.2471/BLT.13.126615 PMC4208475 25378730

[15] Mishra SR, Khanal P, Karki DK, Kallestrup P, Enemark U. National health insurance policy in Nepal: challenges for implementation. Glob Health Action. 2015; 8(1): 28763. DOI: 10.3402/gha.v8.28763 26300556 PMC4546934

[16] Wirtz VJ, Kaplan WA, Kwan GF, Laing RO. Access to medications for cardiovascular diseases in low-and middle-income countries. Circulation. 2016; 133(21): 2076 85. DOI: 10.1161/CIRCULATIONAHA.115.008722 27217433 PMC4880457

[17] Nguyen TA, Knight R, Roughead EE, Brooks G, Mant A. Policy options for pharmaceutical pricing and purchasing: Issues for low-and middle-income countries. Health Policy Plan. 2015; 30(2): 267 80. DOI: 10.1093/heapol/czt105 24425694

[18] World Health Organization. Package of Essential Noncommunicable (PEN) Disease Interventions for Primary Health Care in low-Resource Settings. Geneva: The Organization, 2010. Retrieved from https://www.who.int/nmh/publications/essential_ncd_interventions_lr_settings.pdf (accessed 04 July 2020).

[19] World Health Organization and Health Action International. Measuring medicine prices, availability, affordability and price components 2nd Edition. Geneva: The Organizations; 2008. Retrieved from http://www.who.int/medicines/areas/access/OMS_Medicine_prices.pdf. Accessed 5 Sept 2014.

[20] World Health Organization. WHO/HAI Project on Medicine Prices and Availability [Internet]. 2020. Retrieved from https://www.who.int/medicines/areas/access/Medicine_Prices_and_Availability/en/ (accessed 19 June 2020).

[21] Ewen M, Zweekhorst M, Regeer B, Laing R. Baseline assessment of WHOs target for both availability and affordability of essential medicines to treat non-communicable diseases. PLoS ONE. 2017; 12(2): e0171284. DOI: 10.1371/journal.pone.0171284 28170413 PMC5295694

[22] van Mourik MS, Cameron A, Ewen M, Laing RO. Availability, price and affordability of cardiovascular medicines: a comparison across 36 countries using WHO/HAI data. BMC Cardiovasc Disord. 2010; 10(1): 25. DOI: 10.1186/1471-2261-10-25 20534118 PMC2898673

[23] Penchansky R, Thomas JW. The concept of access: Definition and relationship to consumer satisfaction. Med Care. 1981; 19(2): 127 40. DOI: 10.1097/00005650-198102000-00001 7206846

[24] Wirtz VJ, Moucheraud C. Beyond availability and affordability: How access to medicines affects non-communicable disease outcomes. Lancet Public Health. 2017; 2(9): e390 e1. DOI: 10.1016/S2468-2667(17)30168-8 29253405

[25] Sharma J, Aryal A, Thapa GK. Envisioning a high-quality health system in Nepal: If not now, when? Lancet Glob Health. 2018; 6(11): e1146 e8. DOI: 10.1016/S2214-109X(18)30322-X 30196097 PMC7771520

[26] World Health Organization. HEARTS: Technical package for cardiovascular disease management in primary health care. Geneva: The Organization; 2016. Retrieved from https://www.who.int/cardiovascular_diseases/hearts/Hearts_package.pdf (accessed 13 January 2021).

[27] Management Sciences for Health (MSH). International Medical Products Price Guide, 2015 Edition. Medford, MA: MSH; 2016. Retrieved from https://www.msh.org/resources/international-medical-products-price-guide (25 June 2020).

[28] Zeldin W. Nepal: Minimum Wage Increased. Library of Congress [Internet] 23 5 2016 Retrieved from http://www.loc.gov/law/foreign-news/article/nepal-minimum-wage-increased/ (accessed 20 June 2020).

[29] Mishra SR, Shrestha N, Poudyal IP, et al. May Measurement Month 2017: An analysis of blood pressure screening results in Nepal South Asia. Eur Heart J Suppl. 2019; 21(Suppl D): D83 D85. DOI: 10.1093/eurheartj/suz063 31043887 PMC6479435

[30] Morisky DE, Green LW, Levine DM. Concurrent and predictive validity of a self-reported measure of medication adherence. Med Care. 1986; 24(1): 67 74. DOI: 10.1097/00005650-198601000-00007 3945130

[31] Sison G. The Morisky Medication Adherence Scale: An Overview. [Internet] Seattle, WA: Pillsy; 2018. Retrieved from www.pillsy.com/articles/the-morisky-medication-adherence-scale-definition-alternatives-and-overview (accessed 26 June 2020).

[32] Salam A, Huffman MD, Kanukula R, et al. Two-drug fixed-dose combinations of blood pressure-lowering drugs as WHO essential medicines: An overview of efficacy, safety, and cost. J Clin Hypertens. 2020; 22: 1769 79. DOI: 10.1111/jch.14009 PMC8030031 32815663

[33] Satheesh G, Sharma A, Puthean S, et al. Availability, price and affordability of essential medicines for managing cardiovascular diseases and diabetes: A statewide survey in Kerala, India. Trop Med Int Health. 2020; 25: 1467 79. DOI: 10.1111/tmi.13494 32959441

[34] Mishra SR, Shrestha N, Gyawali B, et al. The changing patterns of Non-communicable diseases and injuries in Nepal from 19902017: A review of evidence from Global Burden of Disease Study 2017. Preprint (Version 1). Research Square. DOI: 10.21203/rs.3.rs-29890/v1

[35] Gyawali B, Mishra SR, Ghimire S, et al. The burden and correlates of multiple cardiometabolic risk factors in a semi-urban population of Nepal: A community-based cross-sectional study. Sci Rep. 2019; 9: 15382. DOI: 10.1038/s41598-019-51454-9 31653888 PMC6814741

[36] Gyawali B, Sharma R, Neupane D, Mishra SR, van Teijlingen E, Kallestrup P. Prevalence of type 2 diabetes in Nepal: A systematic review and meta-analysis from 2000 to 2014. Global Heath Action. 2015; 8(1): 29088. DOI: 10.3402/gha.v8.29088 PMC4662667 26613684

[37] Gyawali B, Hansen MRH, Povlsen MB, et al. Awareness, prevalence, treatment, and control of type 2 diabetes in a semi-urban area of Nepal: Findings from a cross-sectional study conducted as a part of COBIN-D trial. PloS ONE. 2018; 13(11): e0206491. DOI: 10.1371/journal.pone.0206491 30388140 PMC6214524

[38] Ringborg A, Cropet C, Jnsson B, Gagliardino JJ, Ramachandran A, Lindgren P. Resource use associated with type 2 diabetes in Asia, Latin America, the Middle East and Africa: results from the International Diabetes Management Practices Study (IDMPS). Int J Clin Pract. 2009; 63(7): 997 1007. DOI: 10.1111/j.1742-1241.2009.02098.x 19570117

[39] Clark A, Jit M, Warren-Gash C, et al. Global, regional, and national estimates of the population at increased risk of severe COVID-19 due to underlying health conditions in 2020: a modelling study. Lancet Glob Health. 2020; 8(8): E1003 17. DOI: 10.1016/S2214-109X(20)30264-3 32553130 PMC7295519

[40] Kaplan WA, Wirtz VJ, Stephens P. The market dynamics of generic medicines in the private sector of 19 low and middle income countries between 2001 and 2011: A descriptive time series analysis. PloS ONE. 2013; 8(9): e74399. DOI: 10.1371/journal.pone.0074399 24098644 PMC3787029

[41] Khanal S, Veerman L, Ewen M, Nissen L, Hollingworth S. Availability, price, and affordability of essential medicines to manage noncommunicable diseases: A national survey from Nepal. INQUIRY: The Journal of Health Care Organization, Provision, and Financing. 2019; 56: 0046958019887572. DOI: 10.1177/0046958019887572 PMC6906349 31823665

[42] Sharma A, Bhandari PM, Neupane D, Kaplan WA, Mishra SR. Challenges constraining insulin access in Nepala country with no local insulin production. Int Health. 2018; 10(3): 182 90. DOI: 10.1093/inthealth/ihy012 29617832

[43] Adhikari SR, Sapkota DS, Thapa A, Pandey AR. Evaluation of Nepals Free Health Care Scheme from Health System Perspective: A Qualitative Analysis. J Nepal Health Res Counc. 2018; 16(41): 372 7. DOI: 10.33314/jnhrc.v16i41.1584 30739917

[44] Leslie HH, Spiegelman D, Zhou X, Kruk ME. Service readiness of health facilities in Bangladesh, Haiti, Kenya, Malawi, Namibia, Nepal, Rwanda, Senegal, Uganda and the United Republic of Tanzania. Bull World Health Organ. 2017; 95(11): 738 48. DOI: 10.2471/BLT.17.191916 29147054 PMC5677617

[45] Aryal BK, Daud M, Thapa A, Mahotra A, Magar SA, Malla CK. Assessment of health facilities for implementation of package of essential non-communicable disease in Nepal: Baseline study in Kailali and Ilam District. J Nepal Health Res Counc. 2018; 16(2): 149 55. DOI: 10.3126/jnhrc.v16i2.20301 29983428

[46] Hailemichael Y, Hanlon C, Tirfessa K, et al. Catastrophic health expenditure and impoverishment in households of persons with depression: A cross-sectional, comparative study in rural Ethiopia. BMC Public Health. 2019; 19(1): 930. DOI: 10.1186/s12889-019-7239-6 31296207 PMC6625021

[47] Khanal MK, Ahmed MM, Moniruzzaman M, et al. Prevalence and clustering of cardiovascular disease risk factors in rural Nepalese population aged 4080 years. BMC Public Health. 2018; 18(1): 667. DOI: 10.1186/s12889-018-5600-9 29855293 PMC5984400

[48] Singh D, Luz ACG, Rattanavipapong W, Teerawattananon Y. Designing the free drugs list in Nepal: A balancing act between technical strengths and policy processes. MDM Policy & Practice. 2017; 2(1): 2381468317691766. DOI: 10.1177/2381468317691766 PMC6125041 30288415

[49] Thapa R, Bam K, Tiwari P, Sinha TK, Dahal S. Implementing federalism in the health system of Nepal: opportunities and challenges. Int J Health Policy Manag. 2019; 8(4): 195 8. DOI: 10.15171/ijhpm.2018.121 31050964 PMC6499910

[50] World Bank. [Internet]. Washington, DC: World Bank; 2019. New government report takes stock of federalism in Nepal. Retrieved from https://www.worldbank.org/en/news/press-release/2019/12/17/new-government-report-takes-stock-of-federalism-in-nepal (accessed 26 June 2020).

[51] Ranabhat CL, Subedi R, Karn S. Status and determinants of enrollment and dropout of health insurance in Nepal: An explorative study. Cost Eff Resour Alloc. 1 10 2020; 18: 40. DOI: 10.1186/s12962-020-00227-7 33013204 PMC7528465

[52] Ghimire P, Sapkota VP, Poudyal AK. Factors Associated with Enrolment of Households in Nepals National Health Insurance Program. Int J Health Policy Manag. 1 11 2019; 8(11): 636 645. DOI: 10.15171/ijhpm.2019.54 31779289 PMC6885856

[53] Beaney T, Schutte AE, Tomaszewski M, et al. May Measurement Month 2017: An analysis of blood pressure screening results worldwide. Lancet Glob Health. 7 2018; 6(7): e736 e743. 29778399 10.1016/S2214-109X(18)30259-6

