# Supplementary material for: Health System Capacity and Access Barriers to Diagnosis and Treatment of CVD and Diabetes in Nepal
Source: Glob Heart. 2021 May 18;16(1):38. doi: 10.5334/gh.927 (PMC8139299; doi:10.5334/gh.927)
Supplement: Appendix. — Supplementary Online Material. [file gh-16-1-927-s1.pdf]

SUPPLEMENTARY ONLINE MATERIAL

E-Table 1: Summary of published evidence on the 'access' dimensions in Nepal

Authors/ year	Methodology			Outcome measures (Access dimesions)						Findings
	Region and sample size	Surveyed medicines or diagnostics	Study Design	Availability	Price	Affordability	Accessibility	Adoption	Quality	
Devkota et al (2018)	Surveyed 33 PHCs and 13 pharmacies in Nepal	89 formulations for selected NCDs	Cross-sectional (facility) survey	✓	✓	×	×	×	×	Availability ranged from 9·0–88·6%. Price variation was >100% in 37 formulations and <200% in 22 formulations.
Khanal et al (2019)	Surveyed 30 public sector and 30 private sector pharmacies in 6 regions	60 essential medicines for NCDs	Cross-sectional (WHO/HAI facility) survey	✓	✓	✓	×	×	×	Availability was higher in private (78%) than public sector (60%). For all NCDs, patients need to spend <1 day's wage to purchase monthly supply of medicines
Shrestha et al (2017)	Surveyed 94 community pharmacies of Kathmandu valley	8 essential medicines for NCDs (diabetes, CVD, respiratory illnesses)	Cross-sectional (pharmacy facility) survey	✓	✓	✓	×	×	×	Availability was non-uniform (range: 0 – 97%).<br>Affordability ranged from 0·1 – 0·6 days' wages. High price variation and poor access to cost-effective brands.<br>EML was not revised according to disease and drug utilization patterns.
Adhikari et al (2018)	Surveyed 28 public health facilities, 7 district warehouses, and 14 private pharmacies in 7 districts of Nepal	15 essential medicines from WHO's basic list of requirements.	Cross sectional survey	✓	×	×	×	×	✓	Medicine availability was 92·4%. 8·4% of medicines in district warehouses were found to be expired.
Sharma et al (2018)	Surveyed 7 public hospital pharmacies and 35 private pharmacies in Kathmandu Valley. Interviewed pharmacists and wholesalers	Human and analogue insulin products for diabetes.	Cross-sectional (WHO/HAI methodology and qualitative) survey	✓	✓	✓	×	×	×	Mean availability of two insulins listed on EML were 14·3% and 42·9%.<br>The lowest-paid worker would spend 3–17 days' wages to purchase monthly supply.
Shrestha et al (2018)	Interviewed 53 pharmacists or procurement officers of 12 public and 40 private hospital pharmacies in 4 major regions	Procurement practices of all medicines	Semi-structured interviews	×	×	×	×	✓	✓	Majority of hospital pharmacies reported using expensive procurement model for purchasing, without formulary. Procurement was solely based on doctors' prescriptions, influenced by pharmaceutical marketing strategies.

Thapa et al (2016)	Interviewed 234 outpatients from 28 PHCs of 7 districts (3 ecological belts and 5 development regions)	Medicines and health care services for outpatients	Cross sectional facility and patient survey	✓	×	×	✓	×	×	91·2% of prescribed medicines were dispensed.<br>55·6% purchased prescribed medicines from nearby private facilities due to poor public sector availability.<br>40% travelled >30 minutes to reach the facility.
Poudel et al (2017)	Evaluated prescription patterns at primary, secondary and tertiary health care centres in Western Nepal.	Fixed Drug Combinations	Cross sectional descriptive study	×	×	×	×	✓	×	The number of prescription in private centers following EMLs was very low. The cost of utilization of FDCs was higher in private sectors than PHCs. Study found a low use of essential medicines.
Sharma et al (2017)	Evaluated medicine imports as a proxy of medicine availability and price during border blockade (trade embargo)	Retail medicines traded across India-Nepal border during 2015 earthquake blockade period.	Trade database analysis by time series regression.	✓	✓	✓	×	×	×	During blockade, volume of all retail medicines traded across the India-Nepal border was reduced by 46·5%<br>Nepal paid USD 22·3 million more for retail medicines during blockade.
Aryal et al (2018)	Surveyed 92 facilities in Kalaili and Ilam districts	Medicines, procedures and diagnostics for NCDs	Cross sectional (facility) study	✓	×	×	×	×	×	Study reveals the gaps in capacity of health institution and system in training, supply, equipment and diagnostics.
Adhikari et al (2019)	Interviewed healthcare providers and users from 7 districts of Nepal	Medicines and diagnostics under Nepal's free healthcare scheme	Qualitative study with focus group discussion.	✓	×	✓	✓	×	×	Problems in Nepal's free health care include availability of medicines, human resource and diagnostic services. Free health care has improved access to health services specially for poor population
Mishra et al (2015)	Surveyed 3 private and 3 public pharmacies	23 essential cardiovascular medicines	Cross sectional (facility) study	×	✓	×	×	×	×	Price of most medicines in private retail pharmacies were significantly higher than in hospital pharmacies
Bhandari et al (2015)	Interviewed 154 hypertensive patients in Dharan (Eastern Region of Nepal)	Antihypertensive medicines	Cross sectional (patient) study	×	×	×	✓	✓	×	Only 56·5% patients were adherent to antihypertensive medication.<br>The important predictors of non-adherence included high price of medicine (OR 5·14), missed medicine due to cost (OR 0·143)
Leslie et al (2017)	Analysis of data from 716 health centers and 247 hospitals in Nepal	Basic amenities and equipment, infection, essential medicines, diagnostics	Database analysis to calculate service readiness index	✓	×	×	×	×	×	Deficiencies in medicines and diagnostics were particularly common. The readiness index varied between hospitals and health centres/clinics.<br>Most health facilities were insufficiently equipped to provide basic clinical care.
Lim et al (2019)	40 patients and 14 health managers in selected districts with and without Visceral Leishmaniasis (VL) programme.	Diagnostics and surveillance for VL	Cross sectional mixed-method study with structured interviews	✓	×	×	✓	×	×	Diagnostic delay was higher in districts without the VL programme. The main barriers to surveillance included lack of access and awareness in non-program districts and the exclusion of private sector participation in referral, treatment and reporting.
Raffe et al (2013)	75 direct and referral patients	Diagnosis and treatment of	Mixed method study with	✓	×	×	✓	×	×	Those who presented directly to specialist services were 6.6 times more

	from two leprosy hospitals in Nepal	leprosy reactions	qualitative interviews							likely to start appropriate treatment than those presenting elsewhere. Obstacles to early presentation and treatment included diagnostic challenges.
Sunny et al (2020)	Surveyed 814 caregivers for newborns in 11 hospitals in Nepal	Cost of travel, accommodation, treatment (drugs, diagnosis) for newborn care	Cross sectional study	×	✓	×	✓	×	×	Cost of caregivers' stay accounted for more than 40% of the OOPE for sick newborn care, followed by cost of travel, and the baby's stay and treatment. The overall OOPE ranged from 13·6 to 226·1 US dollars (USD).
Shrestha et al (2020)	Surveyed Hospital pharmacies at 2 Nepalese cancer hospitals	31 anticancer medicines belonging to six broad categories	Cross sectional study to assess price variation	×	✓	×	×	×	×	There is wide variation in the price of different brands of anticancer medicines in Nepalese market.

References

- Devkota A, Paudel A, Koirala B, et al. Price variation and availability of free medicine for non-communicable diseases. *J Nepal Health Res Counc*. 2018; 16:118-23.
- Khanal S, Veerman L, Ewen M, et al. Availability, price, and affordability of essential medicines to manage noncommunicable diseases: A national survey from Nepal. *INQUIRY: The Journal of Health Care Organization, Provision, and Financing.* 2019; 56:0046958019887572.
- Shrestha R, Ghale A, Chapagain BR, et al. Survey on the availability, price and affordability of selected essential medicines for non-communicable diseases in community pharmacies of Kathmandu valley. *SAGE Open Med*. 2017; 5:2050312117738691.
- Adhikari SR, Pandey AR, Ghimire M, et al. Universal access to essential medicines: an evaluation of Nepal's free health care scheme. *J Nepal Health Res Counc*. 2018; 16:36-42.
- Sharma A, Bhandari PM, Neupane D, et al. Challenges constraining insulin access in Nepal—a country with no local insulin production. *Int Health*. 2018; 10:182-90.
- Shrestha M, Moles R, Ranjit E, et al. Medicine procurement in hospital pharmacies of Nepal: A qualitative study based on the Basel Statements. *PloS One*. 2018; 13:e0191778.
- Thapa AK, Ghimire N, Adhikari SR. Access to Drugs and Out of Pocket Expenditure in Primary Health Facilities. *J Nepal Health Res Counc*. 2017; 14:139-42.
- Poudel A, Ibrahim MI, Mishra P, et al. Assessment of utilization pattern of fixed dose drug combinations in primary, secondary and tertiary healthcare centers in Nepal: a cross-sectional study. *BMC Pharmacol Toxicol*. 2017; 18:69.
- Sharma A, Mishra SR, Kaplan WA. Trade in medicines and the public's health: a time series analysis of import disruptions during the 2015 India-Nepal border blockade. *Global Health*. 2017;13: 1-9.
- Aryal BK, Daud M, Thapa A, et al. Assesssment of health facilities for implementation of package of essential non-communicable disease in Nepal: baseline study in Kailali and Ilam District. *J Nepal Health Res Counc*. 2018; 16:149-55.
- Adhikari SR, Sapkota DS, Thapa A, et al. Evaluation of Nepal's Free Health Care Scheme from Health System Perspective: A Qualitative Analysis. *J Nepal Health Res Counc*. 2018; 16:372-7.
- Mishra SR, Kandel N, Subedi N, et al. Variation in prices of cardiovascular drugs in public and private pharmacies in Nepal. *Health Serv Res Manag Epidemiol.* 2015; 2:2333392814566508.
- Bhandari B, Bhattarai M, Bhandari M, et al. Adherence to antihypertensive medications: population based follow up in Eastern Nepal. *J Nepal Health Res Counc.*13:3942.
- Leslie HH, Spiegelman D, Zhou X, et al. Service readiness of health facilities in Bangladesh, Haiti, Kenya, Malawi, Namibia, Nepal, Rwanda, Senegal, Uganda and the United Republic of Tanzania. *Bull World Health Organ.* 2017; 95:738-48.
- Lim D, Banjara MR, Singh VK, et al. Barriers of Visceral Leishmaniasis reporting and surveillance in Nepal: comparison of governmental VL-program districts with non-program districts. *Tropical Med Int Health*. 2019; 24:192-204.
- Raffe SF, Thapa M, Khadge S, et al. Diagnosis and treatment of leprosy reactions in integrated services-the patients' perspective in Nepal. *PLoS Negl Trop Dis*. 2013; 7:e2089.
- Sunny AK, Gurung R, Gurung A, et al. Out of Pocket Expenditure for sick newborn care in referral hospitals of Nepal. *Matern Child Health J*. 2020; 24:1-9.
- Shrestha S, Poudel RS, Bhuvan KC, et al. Price variation among different brands of anticancer medicines available in hospital pharmacies of Nepal. *J Pharma Policy Pract*. 2020; 13:1-1

E-Figure 1: Nepal's provinces where the medicine and diagnostics facility survey was conducted

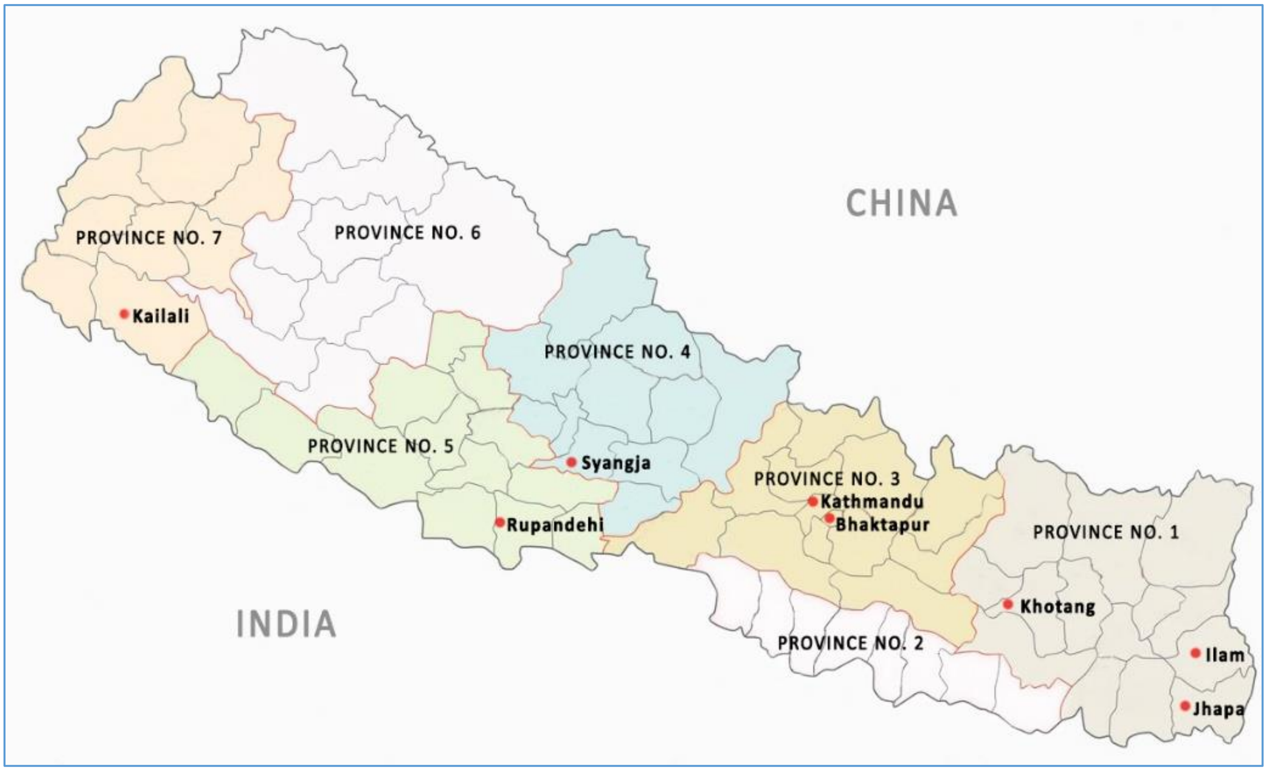

E-Table 2: Median prices of originator brand versions of surveyed medicines

	Originator Brand, Strength and Dosage form	Median unit price of OB (USD)		Median unit prices of generic medicines (USD)		Monthly Need (number of units)	Number of lowest daily wages to pay for monthly supply of OB
		Public sector**	Private sector	Public sector	Private sector		
1	Digoxin *0·25mg tab*	-	0·023	0·0240	0·0225	30	0·34
2	Frusemide *40mg tab*	0.0071	0·0074	0·0077	0·0085	30	0·11
3	Isosorbide Dinitrate *10mg tab*	0.0115	0·0115	-	0·0201	180	1·01
4	Propranalol *40mg tab*	0.0404	0·0355	0·0261	0·0384	120	2·09
5	Spironolactone *25mg tab*	0.0290	0·0297	0·0317	0·0294	90	1·31

Only the medicines for which OB was found are listed in this table
*Lowest daily wage for unskilled workers in Nepal at the time of survey was USD 2·039.
**The median unit price was calculated based on two data points.

E-Table 3: Demographics of patients interviewed in healthcare facility exit survey

**Patient characteristics**	Patients who were diagnosed with and/or prescribed medications for CVD or diabetes (cardio-metabolic) **[N = 636]**
Age (Median, Q1-Q3)	55·0 (44·0–64·0)
**Age group**	
15-24 years	12 (1·9)
25-34 years	48 (7·6)
35-44 years	100 (15·7)
45-54 years	156 (24·5)
55-64 years	168 (26·4)
>=65 years	152 (23·9)
Gender (% Women)	327 (51·4)
**Ethnicity**	
Dalits	63 (9·9)
Disadvantaged Janajati	78 (12·3)
Disadvantaged non-dalit Terai/religious minorities	12 (1·9)
Relatively advantaged Janajati	138 (21·7)
Upper caste group	345 (54·3)
**Region (%)**	
Province-1	22 (3·5)
Province-2,5	59 (9·3)
Province-3	88 (13.8)
Province-4	415 (65·3)
Province-6,7	52 (8·2)
Household size (Median, Q1-Q3)	5·0 (4·0–6·0)
Household income, NPR (Median, Q1-Q3)	25000 (15000–40000) [USD: 240 (144 – 384)]
**Monthly Household income, NPR (USD)**	
<=10,000 rupees (<=USD 95·95)	106 (16·7)
10000-20000 rupees (USD 95·96 - USD 191·90)	168 (26·4)
20000-30000 rupees (USD 191·91 - 287·85)	140 (22·0)
>30000 rupees (>USD 287·85)	222 (34·9)
**BP measured last month (%)**	
Yes	338 (53·1)
No	298 (46·8)
% patients with diabetes	190 (29·9)
% patients with past episode of heart attack	28 (4·4)
% patients with past episode of stroke	17 (2·7)
Systole (Median, Q1-Q3)	130·0 (119·5–141·0)
Diastole (Median, Q1-Q3)	83.8 (77·5–91·5)
Heart rate (Median, Q1-Q3)	78.0 (71·0–86·5)
BMI (Median, Q1-Q3)	24·2 (21·4–27·1)
**Current smoker (%)**	
Yes	106 (16·7)
No	530 (83·3)

Abbreviations: CMDs: cardio-metabolic diseases (defined as CVDs, diabetes or hypertension)

E-Table 4: Adoption of medicines recommended by the WHO in the Nepal national EML

Cardiovascular Medicines	WHO EML 2017	WHO EML 2019	Nepal EML 2011	Nepal EML 2016
Adrenaline, *injection*	✓	✓		✓
Alteplase, *injection*		✓		
Amiodarone*, *oral and injection*	✓	✓		✓
Amlodipine, *oral*	✓	✓	✓	✓
Aspirin, *oral*	✓	✓	✓	✓
Atenolol*, *oral*	✓	✓	✓	✓
Atorvastatin, *oral*			✓	✓
Bisoprolol, *oral*	✓	✓		
Captopril, *oral*			✓	
Carvedilol*, *oral*	✓	✓		
Clopidogrel, *oral*	✓	✓		✓
Digoxin, *oral and injection*	✓	✓	✓	✓
Disopyramide*, *oral*			✓	✓
Dobutamine*, *injection*			✓	✓
Dopamine*, *injection*	✓	✓		✓
Enalapril, *oral*	✓	✓	✓	✓
Fenofibrate, *oral*				✓
Furosemide, *oral and injection*	✓	✓	✓	✓
Glibenclamide*, *oral*	✓	✓	✓	✓
Gliclazide, *oral*	✓	✓		
Glipizide*, *oral*			✓	✓
Glyceryl trinitrate, *sublingual*	✓	✓	✓	✓
Hydralazine, *oral and injection*	✓	✓		✓
Hydrochlorothiazide, *oral*	✓	✓	✓	✓
Intermediate acting insulin, *injection*	✓	✓	✓	✓
Isoprenaline, *injection*			✓	✓
Isosorbide Dinitrate, *sublingual*	✓	✓		✓
Labetalol, *injection*				✓
Lidocaine, *injection*	✓	✓		✓
Lisinopril + amlodipine, *oral*		✓		
Lisinorpil + hydrochlorothiazide, *oral*		✓		
Losartan, *oral*	✓	✓		✓
Metformin, *oral*	✓	✓	✓	✓
Methyldopa, *oral*	✓	✓		
Metoprolol*, *oral*	✓	✓		✓
Nifedipine, *oral*			✓	✓
Prazosin*, *oral*			✓	✓
Procainamide*, *oral and injection*			✓	✓
Ramipril, *oral*				✓
Simvastatin, *oral*	✓	✓		
Sodium nitroprusside*, *injection*	✓	✓	✓	✓
Soluble insulin, *injection*	✓	✓	✓	✓
Spironolactone, *oral*	✓	✓	✓	✓
Streptokinase, *injection*	✓	✓	✓	✓

Telmisartan + amlodipine, *oral*		✓		
Telmisartan + Hydrochlorothiazide, *oral*		✓		
Verapamil, *oral and injection*	✓	✓	✓	✓
Total CVD and diabetes medicines	30	35	23	36

Key takeaways:

1. The 2011 Nepal EML – that would have been in implementation one at the time of our study – had 14 out of 30 (46·7%) CVD and diabetes medicines that were listed in the 2017 WHO Global CEML, and 14 out of 35 (40·0%) listed in the 2019 WHO Global EML.

2. Nepal 2016 EML has 10 medicines [i.e. disopyramide (oral), dobutamine (inj.), Fenofibrate (oral), glipizide* (oral), isoprenaline (inj.), Labetalol (inj.), nifedipine (oral), prazosin (oral), procainamide (oral, inj.), ramipril (oral)] that are not listed on 2017 nor 2019 WHO Global EML.

3. Nepal's national EML was last updated in year 2016 and includes a total of 36 CVD and diabetes medicines (irrespective of dosage form). These include 25 out of 30 medicines (83·3%) listed in the 2017 WHO Global EML, and 25 out of 35 (71·4%) medicines listed in the 2019 WHO Global EML.

4. The 2016 Nepal EML had 4 out of 30 (13·3%) CVD and diabetes medicines that were not recommended in the 2017 WHO Global CEML. These include bisoprolol (oral), carvedilol (oral), gliclazide (oral), and simvastatin (oral).

5. Of the 35 medicines listed in 2019 WHO EML, 9 (25·7%) are not listed in the Nepal 2016 EML. These include Alteplase (inj.), Bisoprolol (oral), Carvedilol (oral), Lisinopril + amlodipine (oral), lisinopril + hydrochlorothiazide (oral), simvastatin (oral), telmisartan + amlodipine (oral), and telmisartan + hydrochlorothiazide (oral).
